# Concurrent production of cellulase and xylanase from *Trichoderma reesei* NCIM 1186: enhancement of production by desirability-based multi-objective method

**DOI:** 10.1007/s13205-017-0607-y

**Published:** 2017-04-08

**Authors:** Preethi Jampala, Satish Tadikamalla, M. Preethi, Swathy Ramanujam, Kiran Babu Uppuluri

**Affiliations:** 1 Bioprospecting Laboratory, Department of Biotechnology, School of Chemical and Biotechnology, SASTRA University, Thanjavur, Tamil Nadu 613401 India; 2 Andaman and Nicobar Centre for Ocean Science and Technology, ESSO-National Institute of Ocean Technology, Port Blair, 744103 India

**Keywords:** Cellulase, Xylanase, *Trichoderma reesei*, *Prosopis juliflora*, Multi-objective optimization

## Abstract

Application of multiple response optimizations using desirability function in the production of microbial metabolites improves economy and efficiency. Concurrent production of cellulase and xylanase in *Trichoderma reesei* NCIM 1186 using an agricultural weed, *Prosopis juliflora* pods, was studied. The main aim of the study was to optimize significant medium nutrient parameters for maximization of cellulase and xylanase by multi-objective optimization strategy using biomass. Process parameters such as the nutrient concentrations (pods, sucrose, and yeast extract) and pH were investigated to improve cellulase and xylanase activities by one factor at a time approach, single response optimization and multi-objective optimization. At the corresponding optimized process parameters in single response optimization, the maximum cellulase activity observed was 3055.65 U/L where xylanase highest activity was 422.16 U/L. Similarly, the maximum xylanase activity, 444.94 U/L, was observed with the highest cellulase activity of 2804.40 U/L. The multi-objective optimization finds a tradeoff between the two objectives and optimal activity values in between the single-objective optima were achieved, 3033.74 and 439.13 U/L for cellulase and xylanase, respectively.

## Introduction

Application of biotechnological process to produce biofuels and value-added chemicals from renewable lignocellulosic biomass had received much attention in recent decades (Erickson and Winters 2012). The key step is the depolymerization of biomass polymers into fermentable sugars. The use of acids/alkalies can be cost efficient but they are not convincible due to their operational high temperature and problematic disposal of acid/alkaline waste. Enzymatic treatment of lignocellulosic biomass offers an efficient and cost-effective hydrolysis at mild conditions. This also avoids additional detoxification steps before successive microbial fermentation. The enzymatic hydrolysis of lignocellulosic biomass typically involves the conversion of cellulose into glucose, and hemicellulose into xylose by cellulase and xylanase, respectively (Zhang et al. 2014). However, enzymatic hydrolysis of lignocelluloses involves high concentrations of both cellulase and xylanase. Interestingly, synergistic effects were reported with cellulases and xylanases which anticipate the fewer enzyme requirements in the simultaneous saccharification of cellulosic biomass (Gonçalves et al. 2015).

Usually cellulase and xylanase were produced and recovered separately from two different fermentation systems. But hemicellulolytic microorganisms such as filamentous fungi produce cellulases and xylanases concurrently with different mechanisms of action (Jun et al. 2011). *Trichoderma, Humicola, Penicillium* and *Aspergillus* species are known to produce hemicellulolytic enzymes in a single fermentation system (Wen et al. 2005; Olsson et al. 2003; Ahamed and Vermette 2010). *Trichoderma reesei* is known to secrete large amounts of cellulolytic enzymes and also utilizes a broad range of carbon sources (Jun et al. 2011; Wen et al. 2005; Esterbauer et al. 1991; Olsson et al. 2003; Ahamed and Vermette 2008, 2010; Domingues et al. 2000).

Synthetic medium ought to be replaced with cheaply and easily available substrates to cut down the enzyme production cost (Manivannan and Narendhirakannan 2014; Liming and Xueliang 2004). Agricultural crop residues and forestry waste containing lignocelluloses serve as both inducer and substrate in the enzymes production and offer great productivity (Szengyel et al. 2000; Lo et al. 2010). Many cellulosic materials such as wood, waste paper, fruit pomace, bagasse, wheat straw, corn cob, wheat bran, aspen wood, waste newsprint, and waste paper sludge have been studied as potential substrates for production of cellulase and xylanase by *T. reesei* (Wen et al. 2005; Reczey et al. 1996; Liming and Xueliang 2004). Thus, there is a great interest in the utilization of cellulosic biomass as a major substrate for the fermentation medium. *Prosopis juliflora* a shrub of semi-arid and tropical part of the world could be used as a fermentative substrate (Shitanda et al. 2014). Due to its high cellulosic content, it offers a very good platform for the growth of microorganisms, especially actinomycetes, and fungi. Recently, we have reported the detailed composition of *P. juliflora* pods (Ramasamy et al. 2014).

Optimization using Response Surface Methodology (RSM) approaches was used to maximize the individual responses of cellulase and xylanase with process variables like carbon source, nitrogen source, pH, temperature and so (Srikanth et al. 2015; Wen et al. 2005; Esterbauer et al. 1991; Olsson et al. 2003; Ahamed and Vermette 2008, 2010; Domingues et al. 2000). But so far there is no report on multi-objective optimization approach to enhance the productivity of cellulase and xylanase simultaneously. Determination of optimal conditions by multi-objective optimization is more effective in certain fermentation systems which yield more than a single response. Especially, the fermentation systems that concurrently produce two products like cellulase and xylanase can be effectively optimized using desirability function at less cost. It also provides more information like the optimal composition of a medium which offers higher yields of desirables than using response surface designs (Kanaga et al. 2016).

In the present work, the multi-objective optimization by desirability function approach was studied using a central composite experimental design (CCD) for obtaining higher cellulase and xylanase from *T. reesei* NCIM1186 in solid–liquid fermentation using pods of *P. juliflora* as a major substrate. Assessment of individual and multi-objective optimization analysis was also performed to compare cellulase and xylanase productivity.

## Materials and methods

### Microorganism and maintenance

*Trichoderma reesei* NCIM 1186 was procured from the National Chemical Laboratory, Pune, India. The organism was cultured and stored on potato dextrose agar (PDA) medium at 4 °C and was subcultured in regular intervals. Glycerol stocks of 80% were prepared and stored at −20 °C for future use. Sporulation inoculum was prepared by adding two loops full of spores from PDA plates into sabouraud dextrose broth and incubated at 30 °C in a shaker for three days and stationary for next three days. Sporulation occurs and this is used as inoculum for all the systems. Inoculum size of 2% v/v containing 2 × 10^4^ spores/mL was used.

### Preparation of substrate

*Prosopis juliflora* pods were collected from the area surrounding SASTRA University campus, Thanjavur, Tamil Nadu, India. Pods were washed under tap water to remove dust and dried under sunlight. Later dried pods were powdered in the grinder and sieved. The particles with the size of 0.42 mm pods were used in the study (Ramasamy et al. 2014).

### Production of cellulase and xylanase from pods containing medium

*Trichoderma reesei* (2 mL, 2 × 10^4^ spores/mL) was inoculated into 250 mL Erlenmeyer flask containing 100 mL sterilized medium (30 g/L pretreated pods and 4 g/L calcium carbonate) at pH 6.5 and incubated at 30 °C for 120 h in a shaker at 150 rpm (REMI CIS24 plus). After the incubation time, the fermented broth was centrifuged at 10,000 rpm at 4 °C for 10 min (REMI C24 plus cooling centrifuge) and enzyme activities were determined in the supernatant.

### Enzyme assay

Cellulase and xylanase activities were assayed by measuring the amount of reducing sugars released from cellulose and xylan, respectively, using dinitro salicylic acid (DNS) assay as described by Ghose et al. (Ghose 1987) and Bailey et al. (Bailey et al. 1992). Briefly, 0.5 mL of culture supernatant was added to 1 mL of 0.05 M citrate buffer of pH 4.8. To this mixture, 0.5 mL 1% w/v carboxymethyl cellulose (CMC) was added as a substrate for cellulase assay and 0.5 mL 1% w/v beech wood xylan was added as a substrate for xylanase assay. All the samples were incubated at 50 °C for 30 min. To this, 2 mL of DNS reagent was added, heated in water bath at 90 °C for 10 min and cooled immediately. Development of color was visible and the absorbance was measured in a spectrophotometer at 540 nm. Reducing sugar concentration was determined using glucose standard for cellulase activity and xylose standard for xylanase activity. Both cellulase and xylanase activities were reported as U/L. One unit of activity was expressed as the amount of enzyme required to release 1 mol of reducing sugar/min under assay conditions.

### Effect of fermentation parameters on the production of cellulase and xylanase by one factor at a time (OFAT)

#### Effect of incubation time, pH, and temperature

To identify the optimum fermentation time, the inoculated medium (pH 6.5) was incubated in a shaker (150 rpm) at 30 °C for 8 days. Samples were withdrawn at every 24 h and enzyme activities were measured as described above. In this OFAT approach, the optimized value of process parameters will be used for the next parameter optimization. Hence, the optimized time will be used for subsequent studies.

To study the effect of pH on the enzyme activities, the prepared media were adjusted to different pH (5.5–8.0) using phosphate buffer or acetate buffer. Media were sterilized, inoculated and incubated, and enzyme activities were measured as described above.

For temperature studies, the inoculated media were incubated at different temperatures (20–45 °C) and enzyme activities were measured as described above.

#### Effect of pods concentration, supplementary carbon and nitrogen sources

The effective concentration of pods for the maximum production of cellulase and xylanase by *T. reesei* was determined by preparing the media with different concentrations of pods (1–6% w/v).

To study the effect of supplementary carbon sources, seven different carbon sources viz., glucose, fructose, xylose, sucrose, maltose, carboxymethyl cellulose (CMC) and lactose were chosen and studied at 0.4% w/v level. Similarly for nitrogen sources, eleven different nitrogen sources viz., peptone, urea, yeast extract, meat peptone, ammonium sulfate, ammonium chloride, ammonium persulphate, potassium nitrate, sodium nitrite, ammonium hydrogen carbonate and casein were chosen and studied at 0.4% w/v level. The selected carbon/nitrogen source was added to the pods containing media.

All the media were sterilized, inoculated and incubated, and enzyme activities were measured as described above.

### Optimization of medium composition for the maximum cellulase and xylanase activity by central composite design

Central composite design (CCD) was adopted for fitting a quadratic surface and to optimize effective parameters and their interactions with 26 numbers of experiments. A regression analysis was performed to model responses, cellulase and xylanase individually. According to CCD, the process variables (4 no.) at five coded levels (−2,−1, 0, 1, 2) were studied with $2^{k} + 2k + n_{0}$ number of treatment combinations, where *k* is the number of independent variables and *n*_0_ is the number of repetition of experiments at the centre point. Dependent variables were enzyme activities (cellulase/xylanase), whereas independent variables were pods (2–6% w/v), sucrose (0–0.8% w/v), yeast extract (0–1.5% w/v) and pH (5.5–7.5). All variable levels *X* were coded as *X*_*i*_ according to the following equation, so that *X*_0_ corresponded to the central value. These experiments were performed in triplicates, and average cellulase (U/L) and xylanase activity (U/L) were analyzed by multiple regressions through least squares method to fit the Eq. (1).

The coded values of the process parameters are determined by the following equation:

1 $${\text{\it{Y}}} = \beta_{0} + \sum \beta_{\text{\it{i}}} {\text{\it{X}}}_{\text{\it{i}}} + \sum \beta_{\text{\it{ij}}} {\text{\it{X}}}_{\text{\it{i}}} {\text{\it{X}}}_{\text{\it{j}}} + \sum \beta_{\text{\it{ij}}} {\text{\it{X}}}_{\text{\it{i}}}^{ 2}$$

where* Y* is the predicted yield, and* β*_0_,* β*_i_, and* β*_*ij*_ are regression coefficients of the model.* X*_*i*_ and* X*_*j*_ represent the independent variables in the coded values. Evaluation of the linear, quadratic and interactive effects of the independent variables on the response was done using the above equation.

### Multi-Objective optimization analysis

In this study, desirability function approach was used to analyze multi-objective optimization for maximization of cellulase and xylanase using Minitab 17.1. The value of each response (cellulase and xylanase) for a given combination of controllable variables was translated to a number between zero and one known as individual desirability. If the maximum value of objective type was obtained, the desirability function would be defined as Eq. (2).

2 $$d = \left\{ {\begin{array}{*{20}l} 0 & \quad {{\text{if }}Y < 1} \\ {\left( {\frac{{Y - L}}{{T - L}}} \right)^{s} } & \quad {{\text{if }}L \le Y \ge T} \\ 1 & \,\quad {{\text{if }}Y > T} \\ \end{array} } \right.$$

where* d* is the desirability function,* L* is the lower acceptable value to the response, *T* is the target value,* Y* is the response and *s* is the weight of the response. Thus, when *s* = 1 (*d* is linear), *s* > 1 (high importance specified near the target value), *s* < 1 (low importance specified near the target value) (Kanaga et al. 2016).

### Models validation

The confirmatory experiment was performed at optimum levels of parameters for maximization of cellulase and xylanase individually as well as a cumulative manner to validate the developed models and kept other medium components level as mentioned in the production medium composition. All model validation experiments and its analysis were conducted in duplicates and average of the result was reported.

## Results and discussion

### Production of cellulase and xylanase

Cellulosic feedstocks and agricultural waste can be used as good fermentative substrates for the production of biochemicals. *P. juliflora* pods containing medium facilitated the growth and metabolism of *T. reesei* NCIM 1186 and yielded a significant level of cellulase, 1714.83 ± 30.02 U/L and xylanase, 209.48 ± 12.78 U/L activities. Few studies were conducted for the concurrent production of cellulase and xylanase from *T. reesei* using different lignocellulosic biomass (Table 1). During the *T. reesei* cultivation in lignocellulosic medium, cellulose and xylan act as inducers for cellulase and xylanase activities, respectively. *P. juliflora* pods are rich in cellulose and contain a low level of hemicellulose and xylan (Ramasamy et al. 2014). Thus, pods cellulose in the medium contributed to the maximum production of cellulase and less hemicellulose may be the reason for the fewer xylanase activities (Zhang et al. 2014). Juhasz et al. (2005) observed the low xylanase production even in the absence of xylan (Juhasz et al. 2005). However, it was evident that the simultaneous production of cellulase and xylanase from the medium containing pods from *T. reesei* has shown promising results when compared with the literature. Therefore, this medium and strain could be tapped further to increase the cellulase and xylanase yields.

**Table 1:** Concurrent production of cellulase and xylanase from *T. reesei* using different lignocellulosic biomass

Substrate	Enzyme activity (U/L)		References
	Cellulase	Xylanase	
*Prosopis juliflora* pods (without optimization)	1714.83 ± 30.02	226.43 ± 12.78	Present study
Switchgrass	720.00	2150.00	Zhang et al. (2012)
Anaerobically digested manure fiber	480.00	1940.00	
Corn Stover	770.00	2140.00	
Water Hyacinth	2319.00	2147.00	Manivannan and Narendhirakannan (2014)
Soybean hulls	360.00	27000.00	Coffman et al. (2014)
Corn stover	930.00	2040.00	Zhang et al. (2014)

### Effect of fermentation parameters on the production of cellulase and xylanase by one factor at a time (OFAT)

#### Effect of incubation time, pH and temperature

**Fig. 1:**
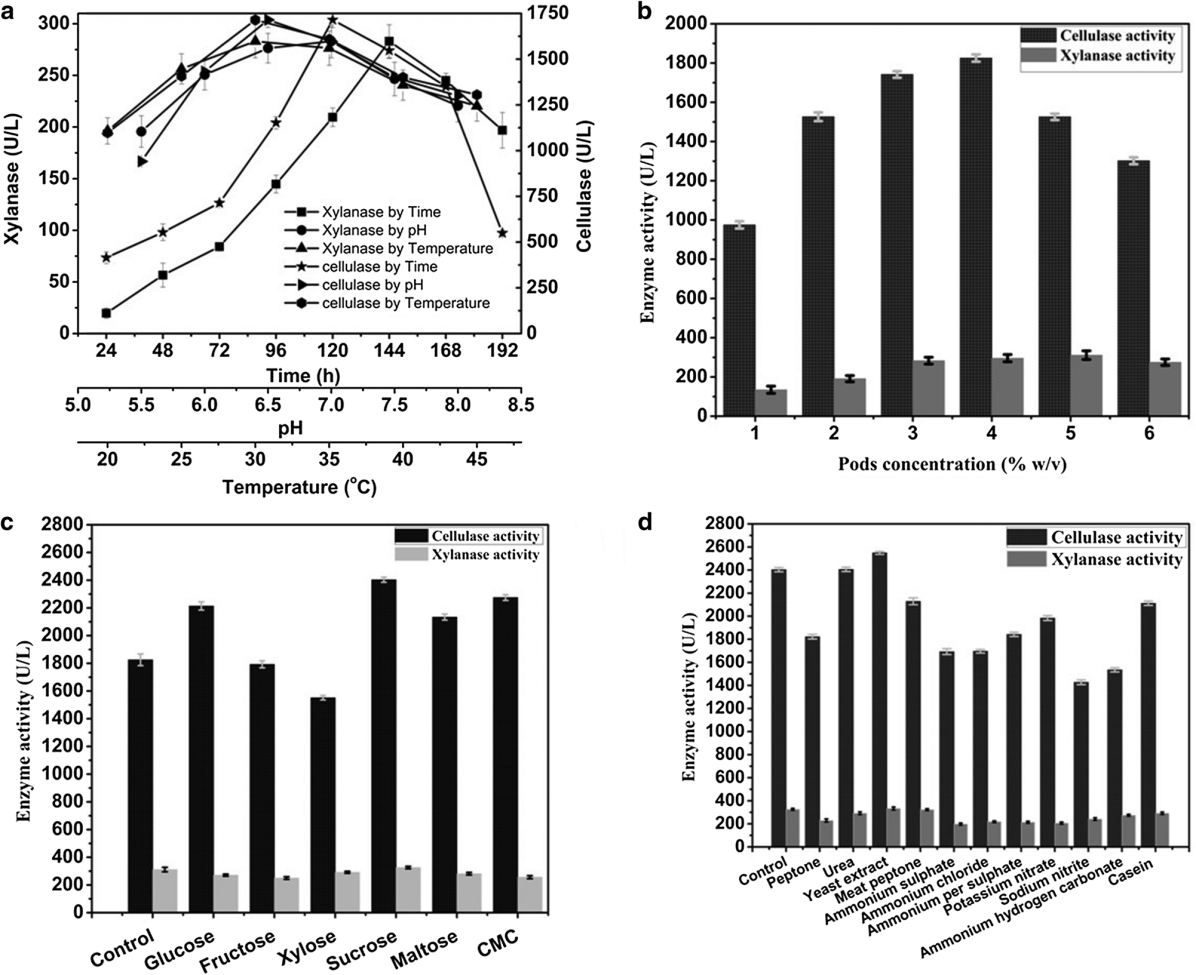
Effect of process parameters on the production of cellulase and xylanase from *T. reesei* using pods containing the medium: **a** effect of time, pH and Temperature, **b** effect of pods concentrations, **c** effect of supplementary carbon sources and **d** effect of supplementary nitrogen sources

Effect of time courses, pH and temperature on cellulase and xylanase production by *T. reesei* in medium containing pods is shown in Fig. 1a. The similar production patterns were observed for both the enzymes but significant variation was noted in enzyme activities. The highest activities were found at 30 °C temperature, 120 h of incubation and 6.5 pH for cellulase; and 30 °C temperature 144 h of incubation and 7.0 pH for xylanase.

Regulation, expression, and formation of depolymerizing enzymes (cellulase, xylanase) in *T. reesei* follow parallel pattern through various control mechanisms (Strauss and Kubicek 1990). Cellulase and xylanase activities were observed from the 24th hour and were continuously increased during first 5 days, reached the maximum levels between 4-6 days and then decreased thereafter. Decrease in the enzyme activities commonly noticed with the inactivation of enzyme secretion systems in a stressed fungal metabolism due to the depletion of nutrients in the fermentation medium (Nochur et al. 1993). The highest levels of enzyme activities were measured during 96–144 h. Similar results have been observed for *T. reesei* with various lignocellulosic biomass (Wen et al. 2005; Esterbauer et al. 1991; Olsson et al. 2003; Ahamed and Vermette 2008, 2010; Domingues et al. 2000).

Temperature and pH are two major process parameters of a fermentation system. Together these will play an important role in inducing the morphological changes in microbes and in enzyme secretion. Especially, the pH change observed during the growth of microbes affects product stability in the medium (Gupta et al. 2003).

Secretion of both the enzymes was observed in the entire range of pH studied but the highest cellulase and xylanase activities were recorded at slightly acidic conditions (pH 6.0-7.0). The highest cellulase activity was found at pH 7.0 and xylanase at 6.5 which is consistent with the previous reports (Jun et al. 2011; Zhang et al. 2012). Filamentous fungi secrete extracellular enzymes under highly regulated mechanisms at pH conditions in which the enzymes are active. In *T. reesei*, ambient pH was shown to be an important determinant of gene expression for biomass and metabolite production (Häkkinen et al. 2015).

Each *Trichoderma* species have their own ecological preferences but most of them had grown better under mesophilic conditions (Papavizas 1985). Several studies pertaining to the optimum temperature for cellulase and xylanase production by *T. reesei* have been reported in the range of 25–30 °C. In the present study, high titers of enzyme activities were found in between 25 and 35 °C (cellulase, 1432.25–1714.83 U/L; xylanase, 256.32–283.04 U/L) with an optimal point at 30 °C, which is in good agreement with the existing literature (Jun et al. 2011; Papavizas 1985; Zhang et al. 2012, 2014).

#### Effect of pods concentration, supplementary carbon and nitrogen sources

As aforementioned, the productions of *T. reesei* enzymes are transcriptionally regulated and depend on the availability of carbon source (Foreman et al. 2003; Juhasz et al. 2005). However, the range of technically applicable substrates is still limited since most of the pure carbon sources are too expensive for industrial fermentations (Jun et al. 2011). Lignocellulosic biomasses have been widely practiced as potential substrates for the fermentative production of industrial enzymes using microorganisms, especially filamentous fungi. Large quantities of *P. juliflora* (leaves, pods and so forth) waste have been generated and are spreading all over the world. Pods and leaves are unpalatable and indigestible; therefore, it is not suitable for animal feed. To overcome the environmental pollution problems linked with the conventional disposal methods, this waste can be used as substrates in fermentations to produce industrially important products with a great economical advantage (Ramasamy et al. 2014; Jampala et al. 2015).

Recently, we have tested the suitability of pods as a substrate for solid–liquid medium for the production of the cellulase by *T. reesei*, but so far no attempts have been made to optimize the enzyme mixture (Jampala et al. 2015). Our results show that medium containing 4% w/v pods serve as an excellent medium for the production of cellulase, 1825.32 ± 42.03 U/L, 5% w/v pods for xylanase, 311.24 ± 15.9 U/L from *T. reesei (*Fig. 1b). Evidently cellulase and xylanase activities were found at even low concentrations of pods, but significant increase (almost 50%) in enzyme activities was seen thereafter. Though pods could serve as a medium for the production of enzymes, the supplementary addition of simple carbon source surely induces the initial growth of *T. reesei* and the production of enzymes. Since, induction of cellulolytic enzymes is more dependent on the carbon source than any other components in the medium (Foreman et al. 2003; Kachlishvili et al. 2006).

Almost 15–25% increased enzyme activities were noted when additional carbon source was given to the medium containing pods. Out of tested carbon sources, sucrose was found to be the best carbon source for both the enzyme productions (cellulase, 2403.29 ± 18.22 U/L; xylanase, 325.62 ± 8.52 U/L) as shown in Fig. 1c. CMC (cellulase, 2274.73 U/L) and glucose (cellulase, 2213.63 U/L) were also shown good cellulase activities. All the tested carbon sources except sucrose have shown a negative impact on the xylanase production. These results suggest that the cellulase and xylanase production by *T. reesei* is carbon source-dependent and that sucrose not only promotes good growth but also efficiently induces the expression of xylanolytic genes (Purkarthofer and Steiner 1995). While sucrose was reported as best supplementary carbon sources for many lignocellulosic biomass (Gautam et al. 2011), CMC (Zhang et al. 2014) and lactose (Jun et al. 2011) also found to be effective for the cellulolytic enzymes by *T. reesei*.

Nature of nitrogen source is one of the potent nutritional factors for regulating the depolymerizing enzymes by filamentous fungi (Sun et al. 2004). The effect of type of nitrogen source depends not only on the fungi physiology but also on the cultivation medium (Kachlishvili et al. 2006). The supplementation of high concentration of organic nitrogen to lignocellulosic medium stimulates the production of cellulolytic enzymes (Kapich et al. 2004). In the present study, 15–25% incremental enzyme activities were recorded when additional nitrogen sources were added to the medium containing pods (Fig. 1d) and the highest enzyme activities were found with yeast extract (cellulase, 2548.91 ± 22.55 U/L; xylanase, 333.38 ± 12.54 U/L). Peptone addition also produced good enzyme activities (cellulase, 2129.13 ± 28.92 U/L; xylanase, 323.07 ± 7.48 U/L). Gautam et al. 2001 also reported the yeast extract and peptone as better nitrogen sources for the production of these enzymes from *T. reesei*.

### Optimization of medium composition for the maximum cellulase and xylanase activity by central composite design (CCD)

With the CCD experimental results, the multiple regression analysis was performed to determine the relationship between tested parameters [Pods (P), sucrose (S), yeast extract (YE) and pH] with measured responses (cellulase and xylanase activities) (Table 2). The significant variation in the activities was observed with the enzymes, cellulase (498.37–3013.45 U/L) and xylanase (2.84–458.03 U/L) indicating the dominant role of selected variables and their concentrations on enzyme production by *T. reesei* NCIM 1186. The low percentage of variation between the observed and predicted values indicates the accuracy of the experiment. The regression coefficients were calculated and data were fitted to second-order polynomial equations for a respective response. The following regression equations demonstrate an empirical relationship between the selected parameters in real values and enzyme activities.

3 $$\begin{aligned} Y_{{{\text{cellulase }}({\text{U}}/{\text{L}})}} &= { 3}0 1 3. 4 4 + { 79}. 60\,\left[ {\text{P}} \right] \, - { 571}. 3 7\,\left[ {\text{P}} \right]*\left[ {\text{P}} \right] \\ & \quad - { 212}.0 6\,\left[ {\text{S}} \right] \, - { 437}. 4 7\,\left[ {\text{S}} \right]*\left[ {\text{S}} \right] \,\\ & \quad - 4 6 1.0 9\,\left[ {\text{YE}} \right]*\left[ {\text{YE}} \right] \, + { 27}0. 8 1\,\left[ {\text{pH}} \right] \, \\ & \quad - { 456}. 4 7\,\left[ {\text{pH}} \right]\\ & \quad *\,\left[ {\text{pH}} \right] \, + { 94}. 7 2\,\left[ {\text{P}} \right]*\,\left[ {\text{S}} \right] \, \hfill \\ & \quad \, + { 1}0 4. 10\,\left[ {\text{P}} \right]*\,\left[ {\text{YE}} \right] \, - { 19}0. 9 6\,\left[ {\text{S}} \right]*\,\left[ {\text{pH}} \right] \hfill \\ \end{aligned}$$

4 $$\begin{aligned} Y_{{{\text{xylanase }}({\text{U}}/{\text{L}})}} = { 434}. 4 2 { } + { 26}. 80\,\left[ {\text{P}} \right] \, - { 33}. 1 4\,\left[ {\text{P}} \right]*\left[ {\text{P}} \right] \, - { 28}. 3 2\,\left[ {\text{S}} \right] \, - { 79}. 70\,\left[ {\text{S}} \right]*\left[ {\text{S}} \right] \, - { 39}. 9 8\,\left[ {\text{YE}} \right] \, \hfill \\ \, - { 7}0. 6 3\,\left[ {\text{YE}} \right]*\left[ {\text{YE}} \right] \, - { 95}. 8 7 { }\left[ {\text{pH}} \right]*\left[ {\text{pH}} \right] \hfill \\ \end{aligned}$$

**Table 2:** Central composite design showing real values along with the observed and predicted cellulase and xylanase activities

Run no	Pods % w/v	Sucrose % w/v	Yeast extract % w/v	pH	Cellulase activity (U/L)		Xylanase activity (U/L)	
					Observed	Predicted	Observed	Predicted
1.	3.00	0.20	0.25	6.00	1243.69	1052.15	159.53	133.52
2.	3.00	0.20	0.25	7.00	2017.09	2003.62	216.24	211.90
3.	3.00	0.20	0.75	6.00	777.81	783.39	166.47	147.68
4.	3.00	0.20	0.75	7.00	1341.41	1417.75	121.50	141.51
5.	3.00	0.60	0.25	6.00	584.49	650.73	136.22	123.84
6.	3.00	0.60	0.25	7.00	967.33	838.34	171.99	139.85
7.	3.00	0.60	0.75	6.00	618.66	721.52	78.75	98.30
8.	3.00	0.60	0.75	7.00	577.69	592.02	21.84	29.77
9.	5.00	0.20	0.25	6.00	634.55	683.04	165.83	188.93
10.	5.00	0.20	0.25	7.00	1989.32	1895.78	356.75	319.52
11.	5.00	0.20	0.75	6.00	692.40	830.70	124.65	139.11
12.	5.00	0.20	0.75	7.00	1729.75	1726.33	141.76	185.16
13.	5.00	0.60	0.25	6.00	727.56	660.54	225.06	187.38
14.	5.00	0.60	0.25	7.00	1052.19	1109.43	205.79	255.61
15.	5.00	0.60	0.75	6.00	1071.45	1147.74	62.50	97.87
16.	5.00	0.60	0.75	7.00	1078.66	1279.52	73.21	81.55
17. (C)	4.00	0.40	0.50	6.50	3013.44	3013.44	410.81	434.42
18.	2.00	0.40	0.50	6.50	498.38	568.78	218.51	248.28
19.	6.00	0.40	0.50	6.50	1029.70	887.17	398.58	355.47
20.	4.00	0.00	0.50	6.50	1634.98	1687.67	172.88	172.26
21.	4.00	0.80	0.50	6.50	964.27	839.44	71.70	58.97
22.	4.00	0.40	0.00	6.50	1021.04	1218.40	186.76	231.87
23.	4.00	0.40	1.00	6.50	1389.24	1119.74	130.42	71.97
24.	4.00	0.40	0.50	5.50	699.46	645.93	12.06	19.93
25.	4.00	0.40	0.50	7.50	1747.78	1729.17	103.20	81.99
26. (C)	4.00	0.40	0.50	6.50	3013.44	3013.44	458.04	434.42

C, experiments were conducted with all the central values of the variables

**Fig. 2:**
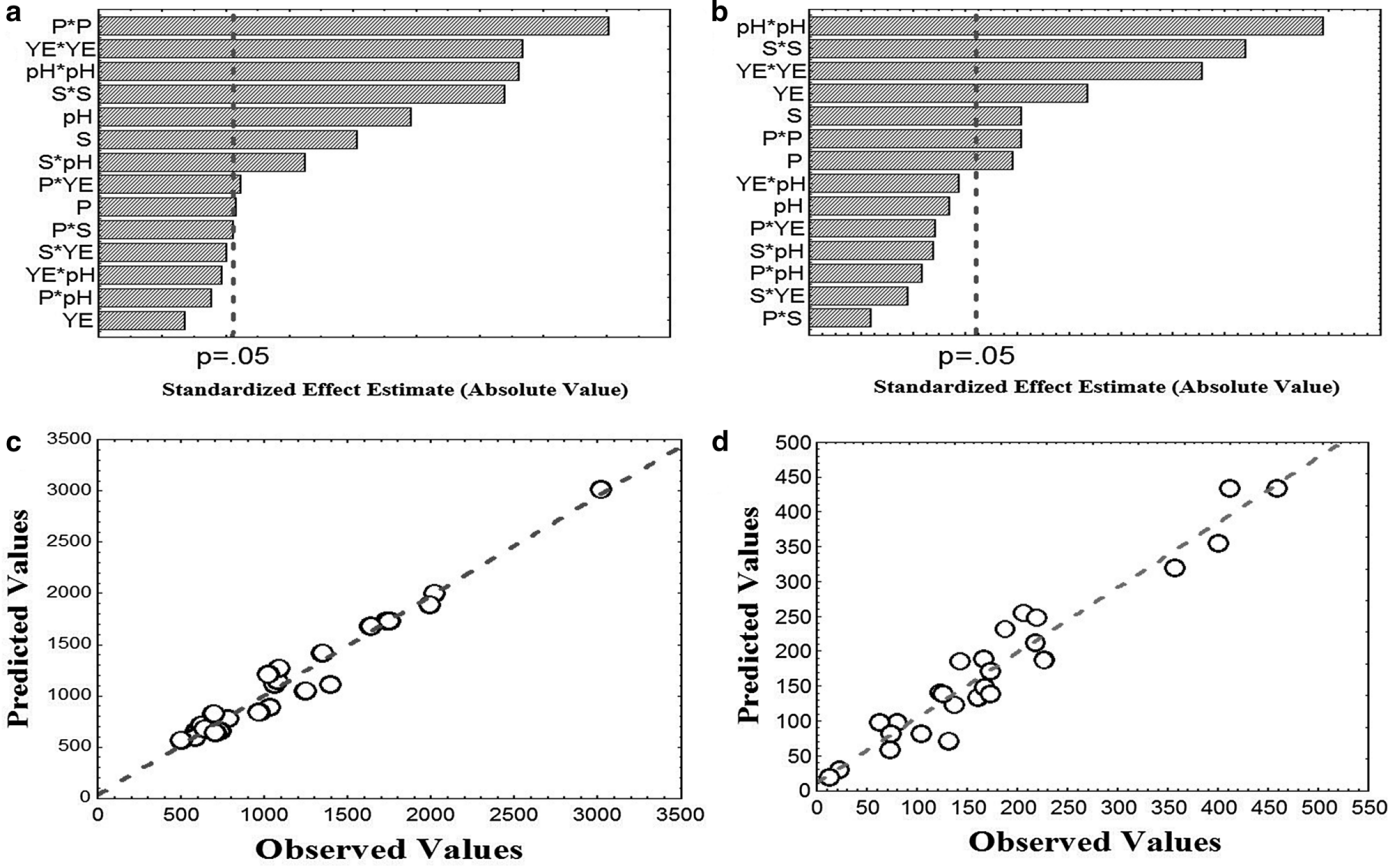
**a**, **b** Pareto chart of effects on cellulase and xylanase activity, respectively, for CCD (*p* = 0.05); **c**, **d** Correlation between the experimental and predicted cellulase (U/L) and xylanase activity (U/L)

The best levels of the selected significant variables were determined for measured experimental responses. Quadratic regression models were developed for cellulase and xylanase using the analysis of variance (ANOVA) (Table 3). The collective effects of all variables for the developed models were contributed to maximizing the cellulase and xylanase activities. The regression coefficients (*R* ^*2*^) were 0.97 and 0.93 for cellulase and xylanase, respectively, indicating that only 2.7% (cellulase) and 6.67% (xylanase) of the variability in the response could not be explained by the model. The high value of adjusted *R* ^*2*^ values (0.94 and 0.85) also suggested the higher significance of models. The goodness of fit for the individual models also indicated that these models for measured cellulase and xylanase were attributed to the tested parameters. The coefficients were selected based on their corresponding *t* and *p* values (Table 3). The overall *p* value of the model is <0.05 for both the enzymes and the *F* value is 197.64 (cellulase), 79.13 (xylanase) (model *F* value  > *p* value), implying that the model is significant. Coefficients which have a low *p* value and high *F* value are considered as significant terms. From the regression analysis, all the quadratic terms of tested variables (P, S, YE, and pH) and linear term of pods and sucrose for both the enzyme activities (cellulase and xylanase) were indicated for their high significance on the basis of their *p* values. Linear terms of pH on cellulase and yeast extract on xylanase have also shown a significant impact. Interactive terms of [P]*[S], [P]*[YE] and [S]*[pH] were highly significant for cellulase activities but no interactive terms are shown significant impact on xylanase activity. Individual significance rankings of linear terms, square terms and interactive terms of variables on cellulase (Fig. 2a) and xylanase (Fig. 2b) were shown in Pareto chart. The correlation plots (Fig. 2c, d) obtained also indicated that the obtained regression model gave a good explanation of the relationship between the independent and response variables. This also indicates an excellent prediction of the response along with parameters value and competence of the developed quadratic models. In the present experiment, the coefficient of variance (CV) was 10.30% cellulase and 4.13% xylanase, which implies good precision and reliability.

**Table 3:** Analysis of variance and model coefficient estimate by multiple regression analysis for cellulase and xylanase activities

	Coeff.		Effect		*t*		*p*	
	Cellulase	Xylanase	Cellulase	Xylanase	Cellulase	Xylanase	Cellulase	Xylanase
Mean/interaction	3013.44	434.42	3013.44	434.42	25.098600	13.645040	0.000000	0.000000
P	*79.60*	*26.78*	159.20	53.59	2.296600	2.915770	*0.042286*	*0.014043*
P*P	−*571.37*	−*33.14*	−1142.73	−66.27	−14.058600	−3.074850	*0.000000*	*0.010568*
S	−*212.06*	−*28.32*	−424.12	−56.64	−6.118400	−3.081600	*0.000075*	*0.010441*
S*S	−*437.47*	−*79.70*	−874.94	−159.40	−10.764100	−7.395490	*0.000000*	*0.000014*
YE	−24.67	−*39.98*	−49.33	−79.95	−0.711700	−4.349550	0.491489	*0.001156*
YE*YE	−*461.09*	−*70.63*	−922.19	−141.25	−11.345300	−6.553420	*0.000000*	*0.000041*
pH	*270.81*	15.51	541.62	31.03	7.813500	1.688070	*0.000008*	0.119511
pH*pH	−*456.47*	−*95.87*	−912.94	−191.73	−11.231600	−8.895430	*0.000000*	*0.000002*
P*S	*94.73*	2.03	189.46	4.06	2.231600	0.180510	*0.047393*	0.860034
P*YE	*104.10*	−15.99	208.21	−31.98	2.452500	−1.420820	*0.032105*	0.183090
P*pH	65.32	13.05	130.64	26.11	1.538700	1.159740	0.152122	0.270707
S*YE	84.89	−9.92	169.77	−19.85	1.999700	−0.881680	0.070839	0.396809
S*pH	−*190.96*	−15.59	−381.93	−31.18	−4.498700	−1.385170	*0.000903*	0.193446
YE*pH	−79.28	−21.13	−158.56	−42.27	−1.867600	−1.877790	0.088670	0.087161
Error								
Total SS								
*R* ^2^	0.97292	0.93331						
Adj *R* ^2^	0.93846	0.84843						

*OFAT* one factor at a time, *CCD* central composite design,* P* pods concentration (% w/v), *S* sucrose concentration (% w/v), *YE* yeast extract concentration (% w/v)

Significant variables were highlighted with italics

**Fig. 3:**
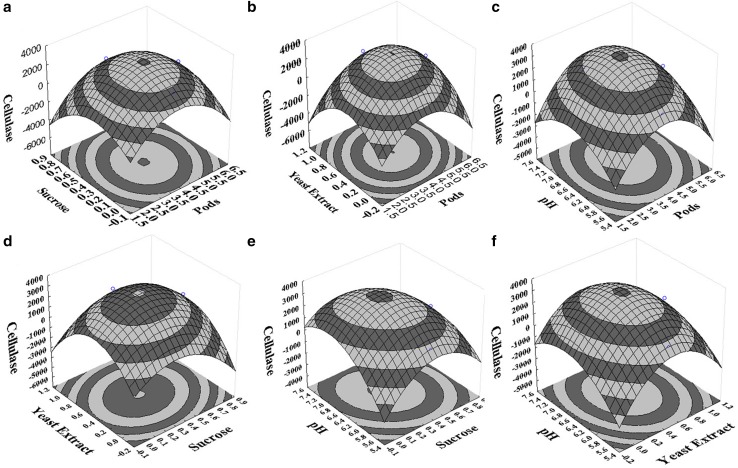
Surface and contour plots of the selected parameters interaction on cellulase activity (U/L): **a** pods concentration (% w/v) with sucrose concentration (% w/v), **b** pods concentration (% w/v) with yeast extract concentration (% w/v), **c** pods concentration (% w/v) with pH, **d** sucrose concentration (% w/v) with yeast extract concentration (% w/v), **e** sucrose concentration (% w/v) with pH, **f** yeast extract concentration (% w/v) with pH

**Fig. 4:**
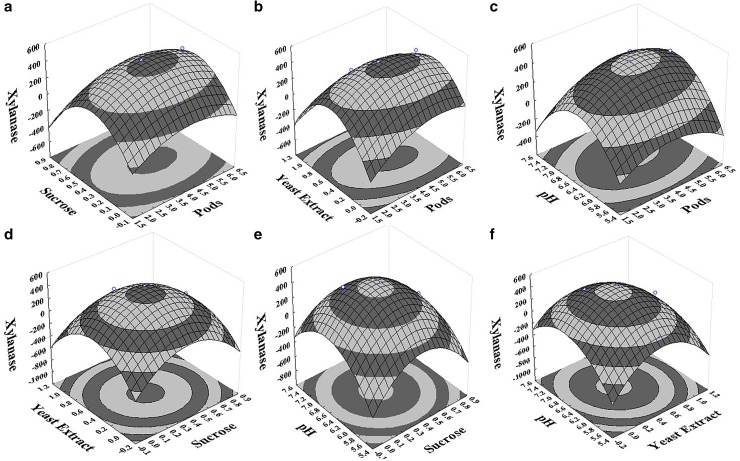
Surface and contour plots of the selected parameters interaction on xylanase activity (U/L): **a** pods concentration (% w/v) with sucrose concentration (% w/v), **b** pods concentration (% w/v) with yeast extract concentration (% w/v), **c** pods concentration (% w/v) with pH, **d** sucrose concentration (% w/v) with yeast extract concentration (% w/v), **e** sucrose concentration (% w/v) with pH, **f** yeast extract concentration (% w/v) with pH

The regression equations (Eqs. 3 and 4) were used to generate 3D and 2D surface & contour plots, respectively (Figs. 3, 4). Using the drawn surface and contour plots interactions, selected variables at different conditions were evaluated. All contours were circular or elliptical in nature, indicating that all selected parameters were independent of each other. Figure 3a–c depicts the interaction of pods with other selected variables on cellulase production and it shows that pods concentration was independent of the pH and slightly dependent on the supplementary carbon and nitrogen sources. Figure 3a, d, e represents the interaction of sucrose with other selected variables on cellulase production. In both Fig. 3a, e, the contours were slightly inclined towards pods and pH indicating that sucrose concentration has a slight influence on the pods concentration and pH of the medium. Figure 3b, d, f represents the yeast extract interaction on the other variables for the cellulase production. Yeast extract has a slight influence on the pods concentration and pH of the medium. Similarly, the xylanase activity 3D and contour plots were observed (Fig. 4a–e). The dependency of pods concentration on the supplementary nutritional source can be clearly seen. Influence of selected variables and their interactions also can be determined from the p values (Table 3). Pods in the range of 3–5% w/v; sucrose, 0.2–0.6% w/v; yeast extract, 0.4–1% w/v; and pH, 6.2–6.8 were effective for both cellulase and xylanase production.

The real concentration of parameters was used into the corresponding regression equations (Eqs. 3 and 4) to predict the maximum output for both the enzyme activities. The maximum cellulase activity (3102.24 U/L) was predicted with the optimized medium components, pods 30.18 g/L, sucrose 2.34 g/L, yeast extract 12.93 g/L and pH 6.5 using *T. reesei* NCIM 1186 where xylanase highest activity was 435.21 U/L. Similarly, the maximum xylanase activity (452.12 U/L) was predicted with medium containing pods 36.4 g/L, sucrose 3.48 g/L, yeast extract 6.56 g/L and pH 6.42 using *T. reesei* NCIM 1186 with highest cellulase activity of 2905.41 U/L (Table 4). Manivannan and Narendhirakannan (2014) applied a Box–Behnken design to optimize carbon (water hyacinth and xylose) and nitrogen (yeast extract and peptone) sources for the enhanced co–production of cellulase (23.19 IU/ml) and xylanase (21.47 IU/ml) by *T. reesei* (Manivannan and Narendhirakannan 2014).

**Table 4:** Comparison of cellulase and xylanase activities of initial, individual and multi-objective optimized medium

Optimization method	Conditions				Enzyme activity			
	Pods (% w/v)	Sucrose (% w/v)	Yeast extract (% w/v)	pH	Cellulase (U/L)		Xylanase (U/L)	
					Predicted	observed	Predicted	observed
OFAT for cellulase	4.00	0.30	0.50	6.50	–	2548.91	–	–
OFAT for xylanase	5.00	0.40	0.40	7.00	–	–	–	333.38
CCD for cellulase	4.05	0.33	0.47	6.60	3102.24	3055.65	435.21	422.16
CCD for xylanase	4.50	0.36	0.41	6.50	2905.41	2804.40	452.12	444.94
Multi-objective	4.14	0.35	0.45	6.60	3078.13	3033.74	445.53	439.13

*OFAT* one factor at a time,* CCD* central composite design

### Multi-Objective optimization analysis

**Fig. 5:**
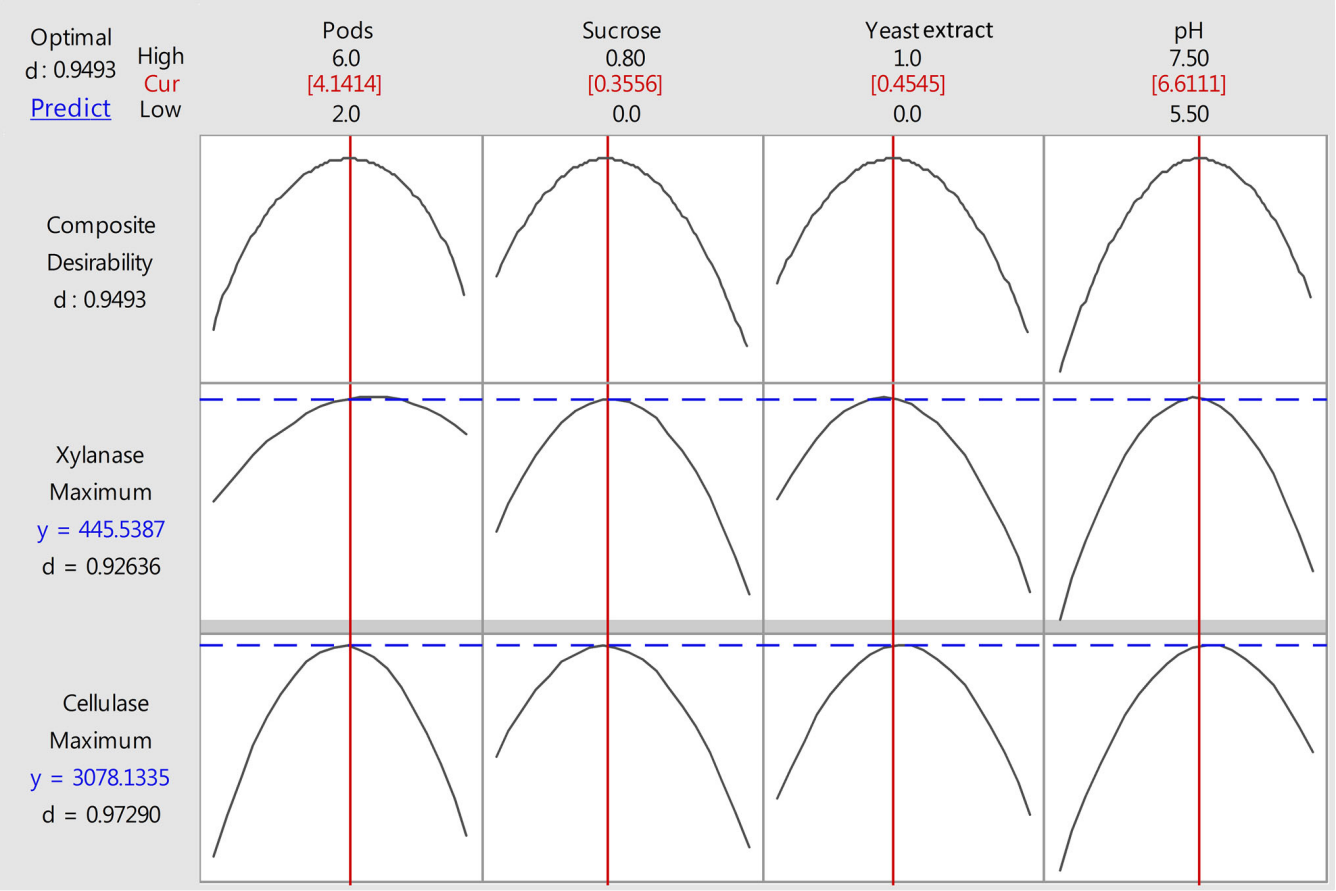
Multi-objective optimization conditions to maximize production of cellulase and xylanase

The multi-objective optimization was performed using desirability function to find out the optimal process parameters that do not adversely affect production of one enzyme type in favor of another. Thus, the optimal tradeoff between production of cellulase and xylanase was achieved (Fig. 5). Results were compared with that of single response variable optimization. The maximum predicted responses obtained by multi-response optimization were cellulase, 3078.13 U/L and xylanase, 445.53 U/L at an optimum level of variables (pods, 41.14 g/L; sucrose, 3.55 g/L; yeast extract, 4.54 g/L; pH 6.6). The optimal enzyme activities obtained from the multi-objective optimization were found to be slightly lower than single response optimized values. However, 20.7 and 33.6% higher value of corresponding cellulase activity and xylanase activities were observed in multi-objective response optimization than one factor at a time optimization.

**Fig. 6:**
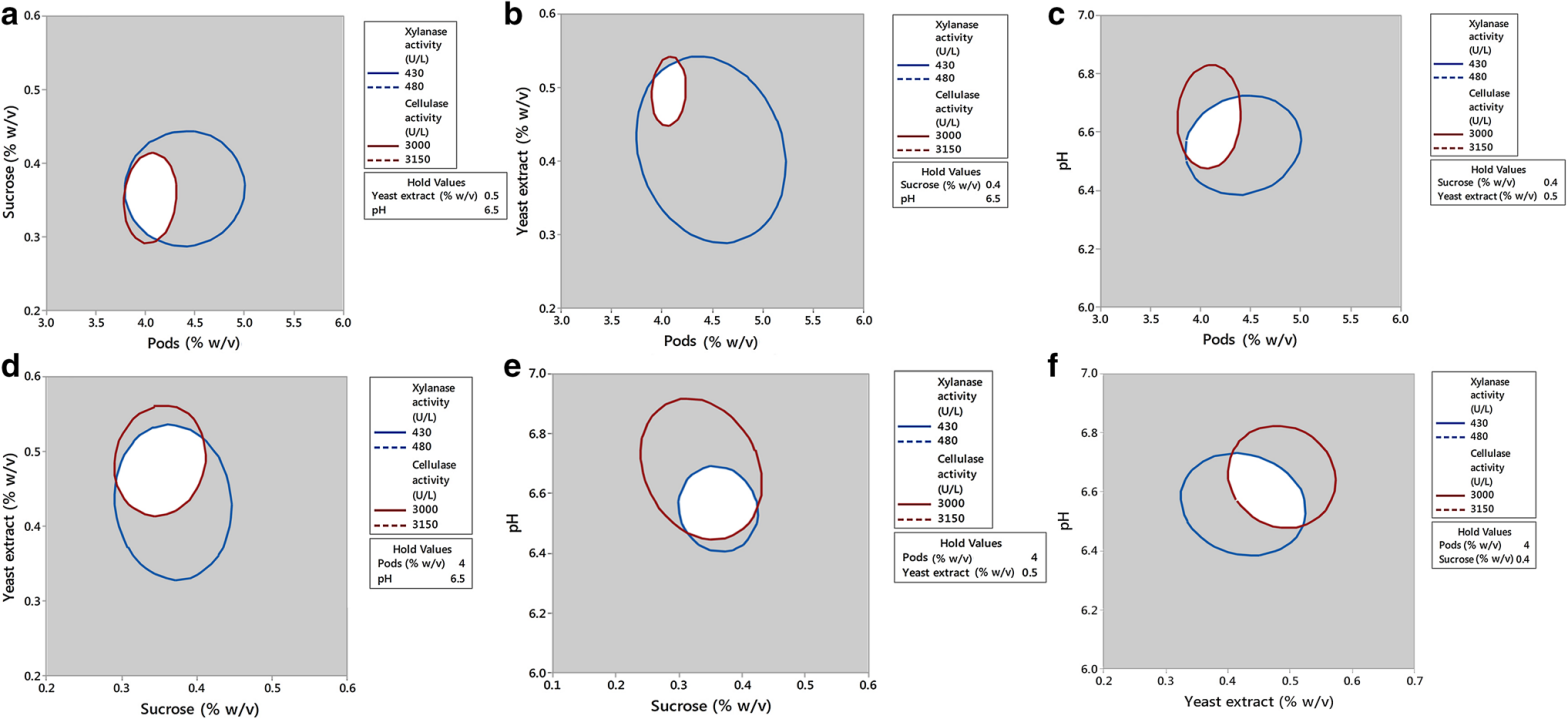
Contour plot showing the maximum response of third variable as mid-value in each plot with varying variables: **a** pods vs sucrose, **b** pods with yeast extract, **c** pods with pH, **d** sucrose with yeast extract, **e** sucrose with pH, **f** yeast extract with pH

The contour surfaces of different responses from important parameters level value can be overlaid and provide the feasible experimental region to fulfill the condition for maximizing the responses altogether. Hence, the overlaid contour plot can be applied to visually elucidate the relationships between the two control factors and two response variables, as shown in Fig. 6. Figure 6a–f corresponds to the overlaid contour diagram for cellulase and xylanase activities from *T. reesei* NCIM 1186 overlapped using Eqs. (3, 4) to represent the optimal experimental region of selected medium components. Although the overlaid contour plot can roughly determine the optimal region for multiple response variables, it is limited to two experimental factors. White region in the overlaid contour diagram indicates the feasible area for maximum values for responses (cellulase and xylanase) under multi-objective optimized conditions. To the best of our knowledge, this is the first report on the multi-objective optimization for cellulase and xylanase.

### Validation of models

To validate the developed models, experiments were performed using modified production media composition with optimal levels of selected parameters while other media components concentration remains the same. Maximization of single responses for *T. reesei* NCIM 1186, the cellulase and xylanase activity was found to be 3055.65 ± 24.23 and 422 ± 16.54 U/L for cellulase production optimized parameters and 2804.40 ± 28.5 and 444.94 ± 16.25 U/L for xylanase production optimized parameters, respectively, which were close to the predicted values obtained in the model. The fitness of single optimization models demonstrated an excellent correlation between predicted and experimental data. The verification of multi-response optimization model had shown a high degree of precision of more than 98.5%. Cellulase and xylanase activities from individual and multi-objective optimized medium are summarized in Table 4. Improvement of single and multi-response optimization approach in comparison with 3% pods containing medium was observed with 19.88 and 19.02% with cellulase activity and 33.33 and 31.72% with xylanase activity correspondingly. The maximum collective responses values at an optimal level were found to be little lower than individual response optimization values. Multi-objective optimization is a more relevant technique than a single response optimization to develop an economic bioprocess which aims multi products.

## Conclusion

Synergistic effect of cellulase and xylanase can be attained for the enhanced lignocellulosic biomass hydrolysis and biofuel production. To make the fermentative production of cellulase and xylanase more economical, the pods of an agricultural weed *P. juliflora* were used as a novel and cheap biomass for the concurrent production of enzymes. High cellulosic content of these pods containing medium facilitated the growth and metabolism of *T. reesei* NCIM 1186 and yielded a significant level of cellulase and xylanase. RSM optimization significantly improved both the cellulase and xylanase observed activities over the ‘one factor at a time’ optimization results. Further, the multi-objective procedure finds an optimal tradeoff between the production of cellulase and xylanase.
